# Estimating blue whale skin isotopic incorporation rates and baleen growth rates: Implications for assessing diet and movement patterns in mysticetes

**DOI:** 10.1371/journal.pone.0177880

**Published:** 2017-05-31

**Authors:** Geraldine Busquets-Vass, Seth D. Newsome, John Calambokidis, Gabriela Serra-Valente, Jeff K. Jacobsen, Sergio Aguíñiga-García, Diane Gendron

**Affiliations:** 1 Instituto Politécnico Nacional, Centro Interdisciplinario de Ciencias Marinas, La Paz, Baja California Sur, Mexico; 2 Biology Department, University of New Mexico, Albuquerque, New Mexico, United States of America; 3 Cascadia Research Collective, Olympia, Washington, United States of America; 4 Marine Mammal and Turtle Division, Southwest Fisheries Science Center, National Marine Fisheries Service, National Oceanic and Atmospheric Administration, La Jolla, California, United States of America; 5 Vertebrate Museum, Department of Biological Sciences, Humboldt State University, Arcata, California, United States of America; University of Alberta, CANADA

## Abstract

Stable isotope analysis in mysticete skin and baleen plates has been repeatedly used to assess diet and movement patterns. Accurate interpretation of isotope data depends on understanding isotopic incorporation rates for metabolically active tissues and growth rates for metabolically inert tissues. The aim of this research was to estimate isotopic incorporation rates in blue whale skin and baleen growth rates by using natural gradients in baseline isotope values between oceanic regions. Nitrogen (δ^15^N) and carbon (δ^13^C) isotope values of blue whale skin and potential prey were analyzed from three foraging zones (Gulf of California, California Current System, and Costa Rica Dome) in the northeast Pacific from 1996–2015. We also measured δ^15^N and δ^13^C values along the lengths of baleen plates collected from six blue whales stranded in the 1980s and 2000s. Skin was separated into three strata: basale, externum, and sloughed skin. A mean (±SD) skin isotopic incorporation rate of 163±91 days was estimated by fitting a generalized additive model of the seasonal trend in δ^15^N values of skin strata collected in the Gulf of California and the California Current System. A mean (±SD) baleen growth rate of 15.5±2.2 cm y^-1^ was estimated by using seasonal oscillations in δ^15^N values from three whales. These oscillations also showed that individual whales have a high fidelity to distinct foraging zones in the northeast Pacific across years. The absence of oscillations in δ^15^N values of baleen sub-samples from three male whales suggests these individuals remained within a specific zone for several years prior to death. δ^13^C values of both whale tissues (skin and baleen) and potential prey were not distinct among foraging zones. Our results highlight the importance of considering tissue isotopic incorporation and growth rates when studying migratory mysticetes and provide new insights into the individual movement strategies of blue whales.

## Introduction

The blue whale (*Balenoptera musculus*) in the northeast Pacific is an endangered migratory mysticete [1]. In summer and fall, blue whales are distributed as far north as the Gulf of Alaska [2,3], but the highest aggregations have been observed off southern California [4]. By mid-fall (~October), they usually migrate south to the west coast of the Baja California Peninsula [2,4–8] and then continue migrating to one of two regions that are recognized as overwintering zones: a calving ground in the Gulf of California [2,9–12], or the Costa Rica Dome in the eastern tropical Pacific [2,7,8]. Calves have also been observed in the Costa Rica Dome, but little is known about the population dynamics in this zone [13].

Blue whales forage throughout their annual migratory cycle mainly on aggregations of krill (Order: Euphausiacea) [14–18] and occasionally on other crustaceans (*i*.*e*. copepods, *Calanus spp*.) [16,19] or small fish (*i*.*e*. lanternfish: Family Myctophidae) [20]. The observation that blue whales forage year-round suggests this species has high energetic demands relative to other migratory mysticetes like the humpback whale (*Megaptera novaeangliae*) and the gray whale (*Eschrichtius robustus*), that typically fast for months during their breeding season in low latitudes [21,22]. The general migratory patterns of blue whales in the northeast Pacific have been described [2,3,5,7,10,11,23], specifically for the California feeding population [3]; however, there are still many gaps in our understanding of their feeding ecology and plasticity in individual movement patterns across multi-year timescales.

Stable isotope analysis (SIA) is a proven tool for studying the diet and movement patterns of marine mammals [24]. The isotopic composition of animal tissues are influenced by diet [25–27] and the isotopic composition of the base of the food web, which can vary in time and space within and among oceanic ecosystems [24,28–31]. Physiological processes produce predictable offsets in isotope values between consumers and their diet, which is often called trophic discrimination [24,32]. In general, consumer tissues have carbon (δ^13^C) and nitrogen (δ^15^N) isotope values that are 0.5–3.0‰ and 2–5‰ higher than that of their prey respectively, depending on the species, diet quality, and type of tissue analyzed [24–26,33,34].

Tissues assimilate dietary inputs at different temporal scales. Most metabolically active tissues reflect recent dietary inputs, consumed within days to months (*e*.*g*. plasma, muscle), depending on their isotopic incorporation rates that typically scale with body mass such that larger animals have slower incorporation rates [35]. In contrast, metabolically inert tissues (*e*.*g*. whiskers, nails) deposit at distinct intervals, and each deposition of tissue retains the isotopic composition of dietary sources incorporated when anabolized, thus reflecting dietary input over several years depending on tissue growth rate [24,36]. Consequently, to make accurate inferences on ecological aspects of free ranging animals by using SIA it is essential to have information on the isotopic incorporation rate of metabolically active tissues and the growth rates of metabolically inert tissues; otherwise, the interpretation of the data can be highly misleading.

SIA of mysticete skin and baleen plates has frequently been used to infer diet and seasonal movements of this difficult to study group of cetaceans [37–44]. Cetacean skin (epidermis) is a metabolically active tissue, subdivided into cellular strata: the stratum basale, the stratum spinosum, and the stratum externum [45,46]. Skin growth begins in the stratum basale a single row of cells that replicate actively. Newly formed cells constantly displace the older cells upward, first to the stratum spinosum, and subsequently to the stratum externum, the outermost layer of skin. Finally, the stratum externum is sloughed off to the environment as sloughed skin [45]. Variation in the isotopic composition among these strata has never been described for any cetacean species. The isotopic incorporation rates of cetacean skin have only been measured in controlled “diet switch” feeding experiments on captive odontocetes [47,48]. These studies used exponential fit models because theoretically, after diet switch, changes in the isotopic composition of tissues will follow an exponential curve over time [49–52]. Estimates of the isotopic incorporation for carbon (δ^13^C) and nitrogen (δ^15^N) in odontocete skin slightly differ; incorporation for δ^13^C is 2 to 3 months, while that for δ^15^N is longer and more variable at 2 to 6 months [47,48]. The increasing use of SIA in mysticetes to characterize diet and movement patterns requires the development of a method to estimate skin isotopic incorporation rates for free-ranging populations.

Baleen consists of a series of keratin plates inserted in the upper gum of mysticetes that functions as a filter-feeding apparatus [53]. In contrast to skin, baleen is a metabolically inert tissue that grows continuously from the gums and abrades at the terminal end [54]. The oscillations in isotope values along the length of baleen plates can be used to estimate growth rates and generate multi-year records of individual movement strategies, habitat use, and diet [38,42–44,55–57]. Baleen growth rates have been estimated in several species of mysticetes [37,42–44,55,57], but currently there are no published estimates for blue whale baleen.

**Fig 1:**
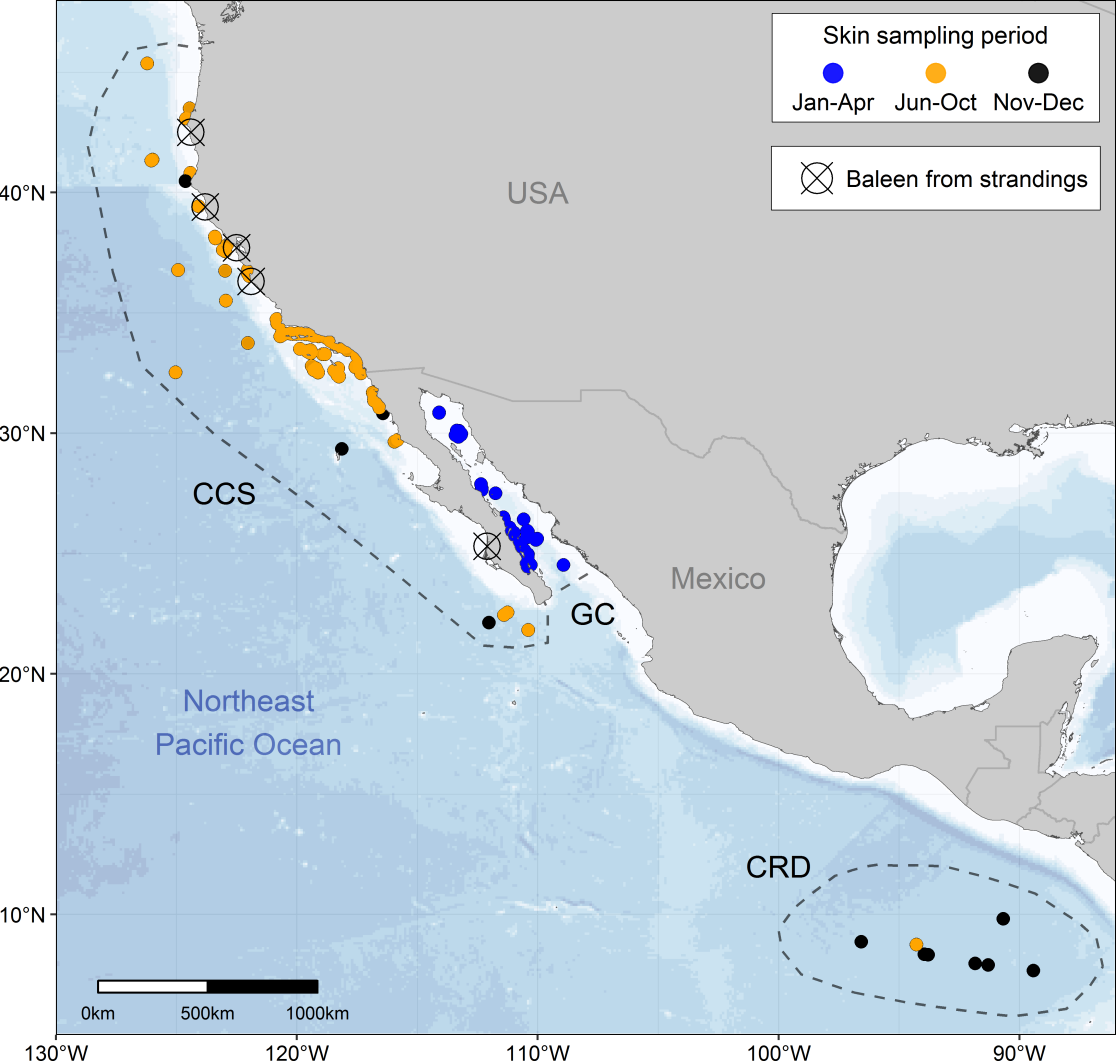
Northeast Pacific sampling zones. Dots represent blue whale skin samples collected in the California Current System (CCS), Gulf of California (GC) and Costa Rica Dome (CRD). Dots with a cross represent blue whale baleen plates collected from dead stranded whales.

Potential prey of blue whales in their distinct summer–fall (California Current System: west coast of U.S. and Baja California Peninsula; Fig 1) and winter–spring (Gulf of California and Costa Rica Dome; Fig 1) foraging zones have contrasting isotope values [58–64] due to differences in oceanographic and biogeochemical processes that influence baseline isotope values in these zones [31,58,62,65]. Specifically, δ^15^N values of prey (*e*.*g*. krill) are higher in the Gulf of California, intermediate in the California Current System, and lowest in the Costa Rica Dome [58–64]. We assumed that blue whale skin strata (stratum basale, stratum externum, and sloughed skin) and baleen plates record these isotopic differences. Then, we evaluated if the seasonal patterns of tissue isotope values could be used to estimate the isotopic incorporation rates and baleen growth rates of blue whale skin and baleen, respectively. We also assessed if carbon isotopes were useful for examining blue whale diet and movement patterns in the northeast Pacific, however, we expected little variation in δ^13^C values of prey among foraging zones based on previous studies [58–64]. Overall, our results highlight the importance of carefully considering the temporal window represented by metabolically active and inert tissues when studying migratory mysticetes.

## Materials and methods

### Ethic statement

All whale tissues used in this study were collected and processed under special permits issued by the Secretaría de Medio Ambiente y Recursos Naturales (SEMARNAT) in México (codes: 180796-213-03, 071197–213–03, DOO 750-00444/99, DOO.0-0095, DOO 02.-8318, SGPA/DGVS-7000, 00624, 01641, 00560, 12057, 08021, 00506, 08796, 09760, 10646, 00251, 00807, 05036, 01110; 00987; CITES export permit: MX 71395), and the National Oceanic and Atmospheric Administration–National Marine Fisheries Service (NOAA/NMFS) (NMFS MMPA/Research permits codes: NMFS-873; 1026; 774–1427; 774–1714; 14097; 16111; CITES import permit: 14US774223/9) in the United States of America. All tissues were collected using non-lethal sampling techniques.

### Sample collection

Blue whale skin biopsies (*n =* 255) and sloughed skin (*n =* 174) were selected from tissue banks at NOAA Southwest Fisheries Science Center (NOAA-SWFSC), Cascadia Research Collective (CRC), and Centro Interdisciplinario de Ciencias Marinas-Instituto Politecnico Nacional (CICIMAR-IPN). These samples were collected from 1996–2015 in the Gulf of California (**GC**) (Jan–Apr; *n =* 115 biopsies, *n =* 81 sloughed skin; Fig 1), California Current System (**CCS**) (Jun–Dec; *n =* 129 biopsies, *n =* 93 sloughed skin; Fig 1) and the Costa Rica Dome (**CRD**) (Oct–Nov; *n =* 11 biopsies; Fig 1). Skin samples were collected during marine mammal surveys conducted by NOAA-SWFSC, CRC, and CICIMAR-IPN. Skin biopsies were collected via dart sampling methods [66], and sloughed skin was directly collected from the water with a net [67] or from suction cups of satellite-tagged whales.

Krill (*n =* 34) and lanternfish (*n =* 7) samples were opportunistically collected during marine mammal surveys conducted by CICIMAR-IPN within the GC (2005–2015). Krill samples were collected by towing a conical net (diameter 50 cm., mesh size 200 μm) when blue whales were observed feeding near the surface. Lanternfish samples were collected with a fishing net (mesh size 5 mm), when aggregations were found near the surface. Prey samples were preserved frozen in liquid nitrogen (-195°C). The assignment of lanternfish to the Family Myctophidae and classification of krill species was made using identification guides [68,69]; *Nyctiphanes simplex* was the only krill species present in all samples.

**Fig 2:**
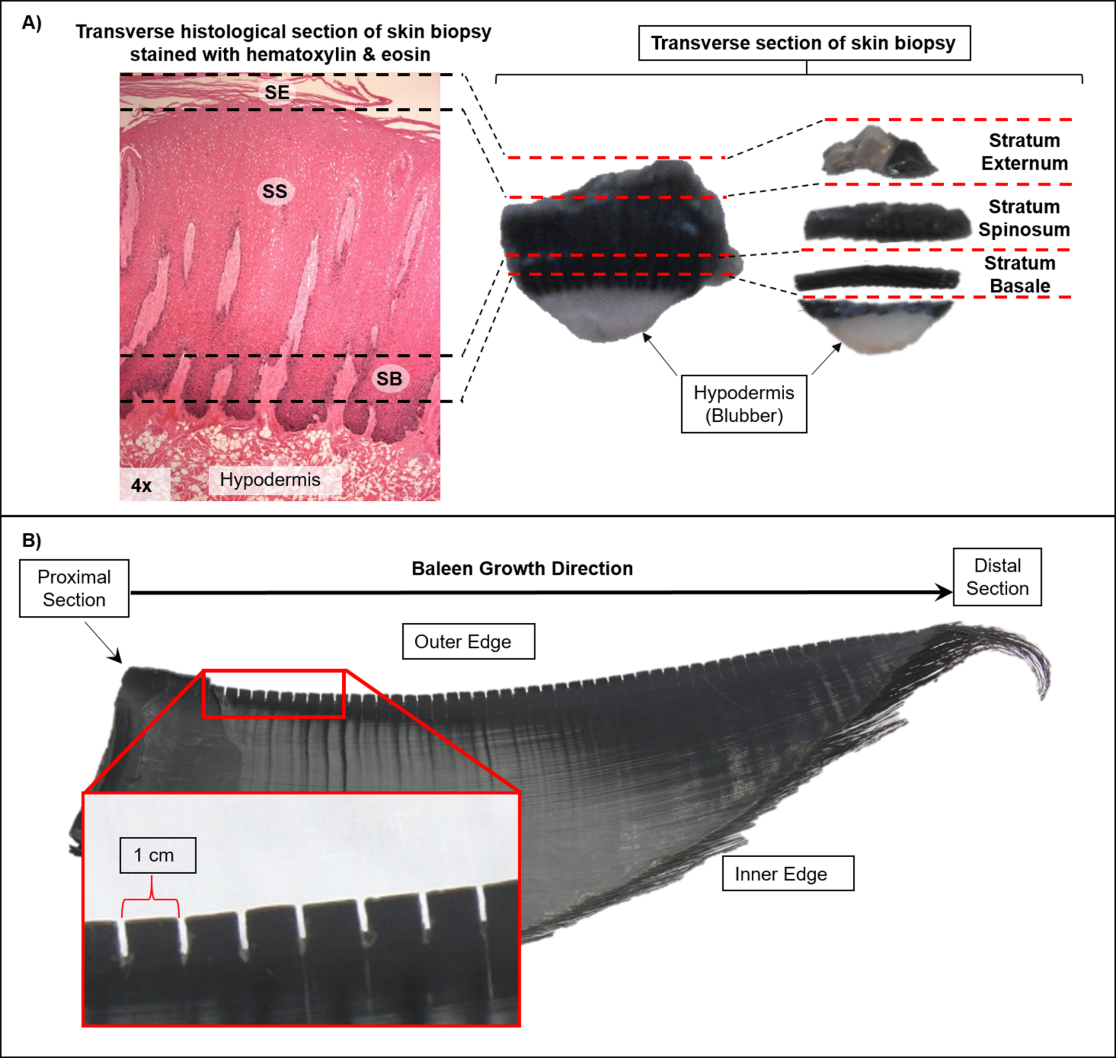
Methods for blue whale skin and baleen plate preparation. (A) Biopsy skin separation into strata: Stratum Basale (SB), Stratum Spinosum (SS) Stratum Externum (SE). The dermal papillae (DP) can be observed embedded in the skin. Dashed lines show were the cuts were made to separate the skin into stratums. (B) Blue whale baleen plate sampling: baleen powder was sub-sampled in 1 cm intervals along the outer edge of the plate starting from the proximal section of the plate nearest the gum.

To assess the isotope variability between blue whale skin strata it was necessary to identify tissue structure. Histological preparations of five skin biopsies were stained with hematoxylin & eosin following the protocol of Sheehan and Hrapchak [70]. Based on these preparations the skin biopsy was divided into two strata: (1) stratum basale, closest to the blubber, and (2) stratum externum, the outermost layer that easily separated from the stratum spinosum (Fig 2A). We did not include stratum spinosum in our analysis because we assumed it would exhibit intermediate isotope values between the stratum basale and the stratum externum. Some skin biopsy samples were incomplete as they had been used for previous studies, and only one of the two strata were available. Sloughed skin samples were also included in the analysis, but were only available for some years (S1 Dataset).

Baleen plates collected from six dead stranded blue whales were obtained from Humboldt State University Vertebrate Museum (HSU-VM), CICIMAR-IPN, the California Department of Parks and Recreation-Prairie Creek Redwoods State Park (CDPR-PCRSP), and the Oregon Marine Mammal Stranding Network (OMMSN) (S1 Table). Stranding reports including sex identification were available for all but one individual, which was determined at NOAA-SWFSC using genetic methods [71,72].

### Standardizing blue whale skin sample preparation

Numerous studies show that two factors that are unrelated to ecology can alter isotope values of metabolically active tissues. The first factor is tissue lipid content. Lipids have lower δ^13^C values than associated carbohydrates and proteins [24,73,74]. Thus, the potential influence of lipid content on bulk tissue δ^13^C values must be considered when using SIA to make ecological inferences [24,75,76]. Chemical lipid-extraction removes the influence of lipids on bulk tissues, but a side effect of this procedure is that it may affect δ^15^N values of tissues [75,76]. To evaluate the effect of lipid-extraction on the isotope values of blue whale skin, five skin samples were divided into two subsamples, one subsample was lipid-extracted with three ~24 hour soaks in a 2:1 chloroform:methanol solvent solution, rinsed with ionized water and lyophilized. The second subsample was simply lyophilized, and analyzed as bulk tissue.

The second factor that can alter tissue isotopic composition is how samples are preserved prior to isotopic analysis. Ideally, all tissues would be stored frozen since freezing does not alter isotope values [24,77–79]. Most of the skin samples selected for this study were stored frozen prior to isotope analysis, but some (*n* = 100) were stored in a 20% salt saturated solution of dimethyl sulfoxide (DMSO). Previous studies have shown that the effect of DMSO on the isotope values of tissues can be removed via lipid-extraction [76,80,81]. To determine if this strategy would work for blue whale skin samples preserved in DMSO, we selected 25 sloughed skin samples from the GC (2005–2007). During field collection, each of these skin samples were divided into two sections and preserved one of two ways for one year before they were prepared for isotope analysis: the first set was preserved in DMSO and the second (control) set was frozen in liquid nitrogen (-195°C).

### Stable isotope analysis

All skin and prey samples were lipid-extracted, lyophilized, and homogenized by grinding them into a fine powder; as noted above the small set of subsamples that were analyzed to test the effects of lipid-extraction were not lipid-extracted (bulk tissue). Baleen plates were cleaned with a solution of 2:1 chloroform:methanol to remove surface contaminants. Sub-samples of keratin powder were collected with a Dremel rotatory drill fitted to a flexible engraving shaft at 1 cm intervals along the outer edge of each baleen, starting at the proximal section inserted in the gum (which represents the newest tissue) (Fig 2B). Baleen grows uniformly on the transverse perspective at a constant (but unknown) rate; thus our sampling strategy would yield equal time intervals between adjacent sub-samples [37,42–44,55,57,82]. Previous studies have confirmed the consistency of isotope values along the length of two adjacent baleen plates of a gray whale (*Eschrichtius robusutu*) [82] and two plates from opposing sides of the mouth of a bowhead whale (*Balaena mysticetus*) [43]. Consequently, we assumed that each baleen provides a consistent record of the past foraging history for each blue whale. Lastly, we compiled δ^13^C and δ^15^N data from the literature of blue whale prey from foraging zones in the northeast Pacific (S2 Table).

Approximately 0.5–0.6 mg of each tissue sample (dried skin, baleen, and prey) was weighed into a tin capsule. Carbon (δ^13^C) and nitrogen (δ^15^N) isotope values were measured with a Costech 4010 elemental analyzer coupled to Thermo Scientific Delta V isotope ratio mass spectrometer at the Center for Stable Isotopes at the University of New Mexico (Albuquerque, NM). Isotope data are reported as delta δ values, δ^13^C or δ^15^N = 1000 [(Rsample / Rstandard)—1], where R = ^13^C/^12^C or ^15^N/^14^N ratio of sample and standard [83]. Values are in units of parts per thousand or per mil (‰) and the internationally accepted standards are atmospheric N_2_ for δ^15^N and Vienna-Pee Dee Belemnite limestone (V-PDB) for δ^13^C [83]. Within-run analytical precision was estimated via analysis of two proteinaceous internal reference materials, which was ±0.2‰ for both δ^13^C and δ^15^N values. We also measured the weight percent carbon and nitrogen concentration of each sample and used the C/N ratio as a proxy of lipid content [84].

### Statistical analysis

All statistical analyses were performed using R [85]. The effects of preservation (DMSO-lipid extracted *vs* frozen-lipid extracted) and the different treatments (lipid-removal *vs* bulk tissue) on skin δ^13^C, δ^15^N and C/N ratios were evaluated with a max-*t test* for multiple comparisons of means. This procedure was chosen because it is designed to work in scenarios of unbalanced group sizes, non-normality and heteroscedasticity [86]. The isotopic variability between skin strata (basale, externum, sloughed skin) was also evaluated by using the max-*t test*, which has a higher power to detect differences between group means compared to other methods [86]. These analyses were performed separately for each zone (GC and CCS) and isotope (δ^13^C or δ^15^N). The CRD skin isotope values were excluded from this analysis as sloughed skin samples were not available for this zone.

The prey data were used to establish the reference mean (±SD) baseline isotope values within each zone, hereafter called the prey zone mean, which was estimated by pooling the means and variances of all the data. The pooled prey zone mean for the GC included lanternfish and the krill species *Nyctiphanes simplex*, because molecular analysis of fecal samples has shown that blue whales forage only on combined aggregations of both taxonomic groups in this zone [14,20]. Lanternfish was the only teleost fish present in blue whale fecal samples [20]. In the CCS, we included isotope values of its main prey, the krill species *Thysanoessa spinifera* and *Euphausia pacifica* [15,18]. In the CRD, diving behavior and the presence of whale fecal samples confirmed that blue whales forage on patches of krill [17], however, the species of krill was not identified, so we used previously reported data for krill in this zone [62].

Our approach to estimate the blue whale skin isotopic incorporation rate was to mimic a diet switch in controlled feeding experiments, but at population level (sampling the same individual whale across its annual migratory cycle is logistically impossible). Blue whales in the northeast Pacific are ideal for this approach because they feed year-round and seasonally migrate between zones that have distinct baseline isotope values [28,31,58,62,64,87]. To achieve this, first we evaluated if blue whale skin δ^13^C and δ^15^N values exhibited seasonal trends in the GC (Jan-Apr) and the CCS (Jun-Dec). Sampling effort within each zone was not homogeneous for all years, thus blue whale skin samples collected in different years were integrated into a single analysis. We assessed the seasonal trend by fitting a generalized additive model (GAM) of the skin δ^15^N and δ^13^C values as functions of time (Julian day, which ranges from 1 to 365). This was done separately for each skin stratum (basale, externum, and sloughed skin) in both foraging zones (GC and CCS). We used GAMs because they are especially useful when the functional form of the relationship between the response (*e*.*g*. δ^15^N and δ^13^C values) and explanatory variables (*e*.*g*. time) is unknown [88]. GAMs were fitted using the “mgcv” package in R [85,89]. To model the main trend of the data, the smoothing parameters (degrees of freedom) were set to three. This conservative approach can be applied when sample size is low [90]. Blue whale skin strata δ^13^C did not show seasonal trends (see Results and S1 Fig), therefore, the isotopic incorporation rate was only estimated for skin δ^15^N.

To compare the δ^15^N values of the three skin strata to potential prey, we assumed a trophic discrimination factor (Δ^15^N) of 1.6‰, based on controlled feeding experiments on captive bottlenose dolphins (*Tursiops truncatus*) [47,48], and calculated the trophic-corrected mean blue whale skin values for each zone by adding this trophic discrimination factor to the prey zone mean values. These trophic-corrected skin values would represent the expected mean δ^15^N values if blue whale skin had fully equilibrated with that of local prey (or reached steady-state isotopic equilibrium), and we assumed that this method would allow us to assign any given blue whale skin isotope value to a specific foraging zone.

Based on the gradient in the prey mean isotope values for each foraging zone (GC>CCS>CRD; S2 Table), and the trophic-corrected blue whale skin values (see Results), our hypothesis was that blue whales would arrive to the GC with lower skin δ^15^N values due to consumption of prey in the CCS and CRD. Skin isotope values would then increase throughout the winter season as they equilibrate with local prey (see Results). In contrast, most whales would arrive in the CCS with higher skin isotope values, except for individuals that migrated from the CRD. Thus, we predicted that skin isotope values would decrease throughout the summer season as skin isotopically equilibrated with the local prey in the CCS. Therefore, we used the GAMs seasonal predictions to estimate the isotopic incorporation rate for each skin stratum, as the days that it would take for the skin δ^15^N to increase (GC) or decrease (CCS) by the assumed trophic discrimination factor (Δ^15^N = 1.6‰) to reach isotopic equilibrium with the local diet. This period was derived by extrapolating from the distance between the predicted extremes in δ^15^N for each stratum, from the lowest to the highest in the GC and vice versa for the CCS (S3 Table, S2 Fig). In this case, we assumed that the equivalent to the diet switch stage would be the lowest initial δ^15^N value within the GC and the highest initial δ^15^N value in the CCS (S2 Fig). We used the same method with the 95% upper and lower confidence intervals to assess uncertainty (S3 Table, S2 Fig). Unfortunately, the uncertainty associated to individual variability in isotopic incorporation rates given the potential variation in individual arrival and departure times to/from the GC and CCS, could not be considered in the model.

Due to sample size limitations, we had to integrate all the skin data collected in different years into a single seasonal model to estimate blue whale δ^15^N isotopic incorporation rate. This assumes that the relative difference in prey δ^15^N values between foraging zones is consistent across years, which has been suggested in previous studies [58,91]. We evaluated this assumption by fitting a generalized linear model (GLM) of skin δ^15^N values as a function of time (Julian Date, or date of sample collection). Julian Dates are a continuous count of days based on a standard starting point, which we chose as January 1, 1970 (Universal Time, Coordinated). This analysis was made separately for each foraging zone (GC, CCS and CRD) by using all skin strata, which allowed us to evaluate the trends in skin δ^15^N across years in each zone. The GLMs were fitted by using the “glm” function in R [92].

Oscillations in δ^13^C and δ^15^N values of baleen plates were also evaluated with a GAM model and smoothing parameters were selected by standard data-driven methods for time series using Akaike Information Criteria [93,94]. Similar to skin, baleen δ^13^C values were not distinct among foraging zones (see Results and S3 Fig), consequently growth rates were estimated using δ^15^N values. Blue whale baleen growth rate was determined by assuming that the oscillation in δ^15^N values along the total length of the outer edge of the baleen plates represent the annual movement between winter/spring and summer/fall foraging grounds. Thus, the distance between two sequential δ^15^N minimums represents the growth of the baleen plate during a single year [37,42–44,55]. Additionally, to characterize the movement of whales among isotopically distinct foraging zones, we compared baleen δ^15^N values with the trophic-corrected δ^15^N values for each foraging zone based on the same Δ^15^N used in the skin analysis [47,48].

## Results

Blue whale skin isotope values are available in S1 Dataset. The max-*t* test results comparing the effect of different treatments (bulk tissue *vs* lipid-extracted; frozen *vs* DMSO) on skin δ^15^N, δ^13^C and C/N ratios are presented in S4 Table. Lipid-extracted skin (-16.5±0.1) had mean δ^13^C values that were significantly higher (1.9‰) than bulk skin samples (-18.4±0.4; *t* = -10.4, *p* = <0.001), and the weight percent C/N ratios of bulk skin were significantly higher (4.2±0.1) than lipid extracted samples (3.2±0.0; *t* = 12.9, *p* = <0.001). In contrast, skin δ^15^N values did not differ significantly between lipid-extracted (14.6±0.3) and bulk skin (14.5±0.3; *t* = -0.4, *p* = 0.7). Lastly, δ^15^N, δ^13^C, and C/N ratios of skin samples stored in DMSO (δ^15^N: 13.9±0.9; δ^13^C: -16.9±0.5; C/N: 3.0±0.2) did no differ significantly from skin stored frozen (δ^15^N: 14.0±0.9; δ^13^C: -16.9±0.6; C/N: 3.0±0.2); δ^15^N: *t* = 0.2, *p* = 0.8; δ^13^C: *t* = 0.2, *p* = 0.8; C/N: *t* = -0.4, *p* = 0.7.

The max-*t* test results comparing the δ^15^N and δ^13^C values among skin strata (basale, externum and sloughed skin) in each zone (GC and CCS) are shown in S5 Table. Skin δ^15^N and δ^13^C did not differ significantly between different skin strata within the GC (S5 Table). In the CCS, mean δ^15^N values of sloughed skin (13.6±0.7‰) and stratum externum (13.4±1.1‰) did not differ significantly (*t* = -0.4, *p* = 0.7), and both of these strata had slightly but significantly higher δ^15^N (stratum externum: *t* = 2.6, *p* = <0.001; sloughed skin: *t* = -4.9, *p* = <0.001) than the stratum basale (13.0±0.8‰). δ^13^C values did not differ significantly among strata in the CCS (S5 Table).

The GLM model of blue whale skin δ^15^N values as a function of time (Julian Date) was not significant in the CRD (1999–2003; S6 Table, S4 Fig). Conversely, the relationship between these variables was significant and positive in the GC and the CCS (S6 Table, S4 Fig). The GLM model predicts an overall increase of 1.2‰ over 15 years (1996–2011) in the CCS, and an increase of 0.8‰ over 13 years (2002–2015) in the GC (S6 Table, S4 Fig); overall, these shifts results in a 0.1‰ increase per year in each zone. Thus, skin δ^15^N values showed a slight and consistent trend in both zones, therefore the gradient in δ^15^N values between zones would also remain constant. This result would validate the integration of blue whale skin δ^15^N values in a single seasonal GAM model to infer skin δ^15^N isotopic incorporation rate for each zone.

### Skin isotopic incorporation and baleen growth rates

**Fig 3:**
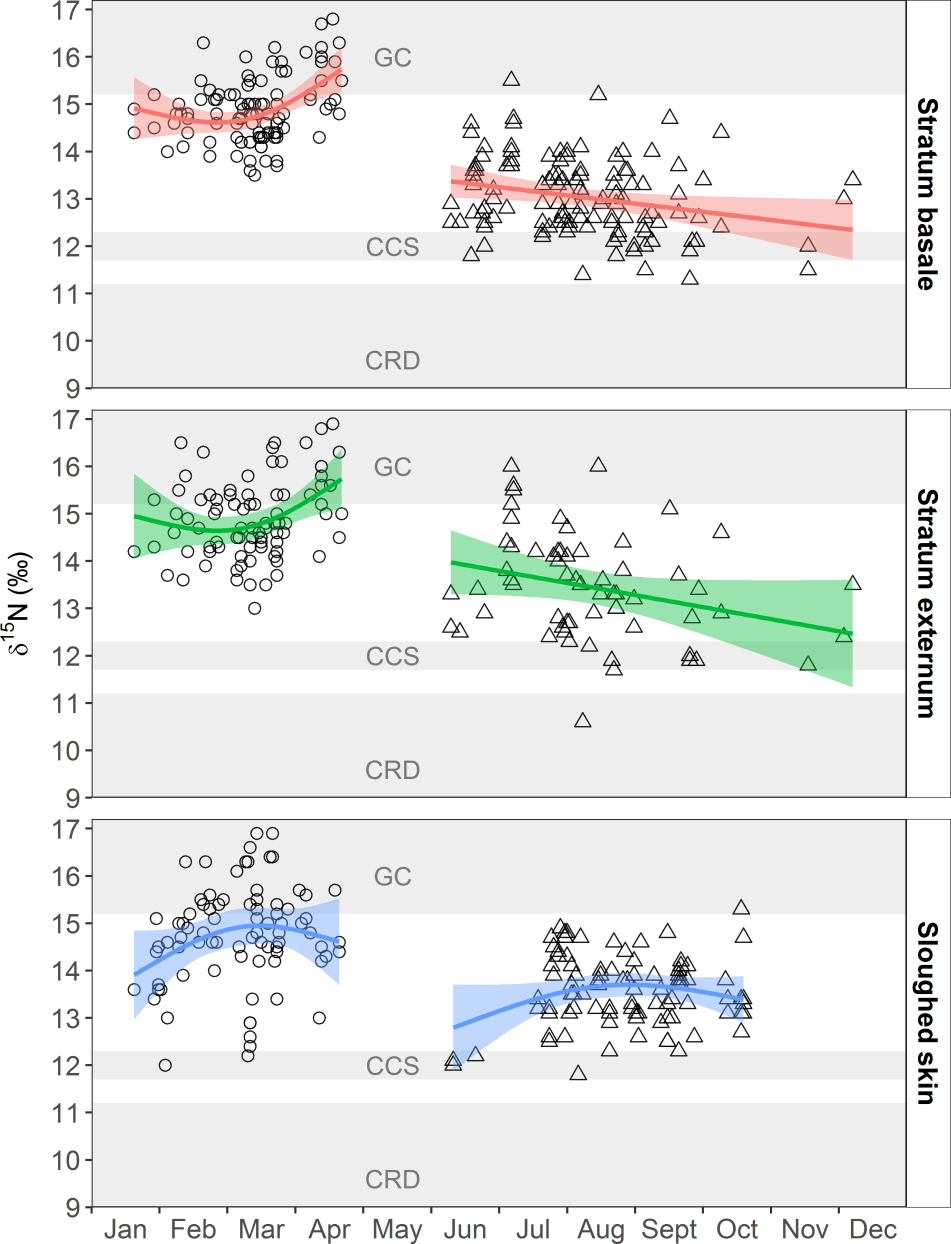
GAM analysis of the seasonal trend of skin strata δ^15^N values in two foraging zones. The points represent the actual δ^15^N values of skin collected from whales within the Gulf of California (open circles) and the California Current System (open triangles). The colored lines represent the GAM model fit (predictions) and the fringe around the lines show the 95% confidence intervals. The gray shaded area represents the mean±SD of the trophic-corrected blue whale skin values for each foraging zone: Gulf of California (GC), the California Current System (CCS) and the Costa Rica Dome (CRS).

Prey from the three zones had distinct δ^15^N values (S2 Table), with values decreasing from the GC to the CCS and CRD. The trophic-corrected blue whale skin δ^15^N values for each foraging zone are presented in Table 1. The magnitude of differences in prey between these zones ranged from 1.9‰ to 6.1‰ (S2 Table), which allowed us to assign the origin of measured δ^15^N values of the different blue whale skin strata, independently of the zone where whales were sampled (Table 1, Fig 3).

**Table 1:** Trophic-corrected blue whale skin δ^15^N values for each foraging zone.

Zone	Prey zone mean (±SD) δ^15^N	Δ^15^N	Trophic-corrected blue whale skin δ^15^N
Gulf of California	14.6±1.0	1.6	16.2±1.0
California Current System	10.4±0.3	1.6	12.0±0.3
Costa Rica Dome	8.5±1.1	1.6	10.1±1.1

Values were estimated by using the prey zone mean±SD (S2 Table) and assuming Δ^15^N of 1.6‰.

The GAM results of the relationship between blue whale skin δ^15^N values and time (seasonal trend) are shown in Table 2. The GAM that used δ^15^N values in blue whale skin stratum basale and externum in relation to time indicated a weak, but slightly significant positive relationship in the GC, and a weak, but slightly significant negative relationship for the CCS (Table 2, Fig 3). These relationships were anticipated based on the observed pattern in prey δ^15^N values among zones and the trophic-corrected blue whale skin values for each foraging zone (Table 1, S2 Table). For samples collected in the GC, δ^15^N values increased to ~17‰ by April (Fig 3), which likely reflected isotopic equilibration with the δ^15^N of local prey (Table 1). The opposite pattern was observed in the CCS, were the δ^15^N values decreased with time to a low of ~13‰ by December (Fig 3), which also suggests gradual equilibration of the tissue to the local prey. In contrast, the relation between sloughed skin δ^15^N values and time was not significant in the GC or CCS (Table 2). The GAM model for sloughed skin showed a parabolic relationship with time, with a slight tendency of the δ^15^N values to increase and subsequently decrease with time in both zones (Fig 3). Therefore, we used the same method than that for the stratum basale and externum within each zone to estimate the isotopic incorporation rate of sloughed skin (S2 Fig).

**Table 2:** GAM results for the seasonal trends of δ^15^N and δ^13^C values in different skin strata sampled in the Gulf of California (GC) and California Current System CCS).

Isotope	Skin stratum	Zone	n	*E*.*df*.	F	Adjusted R^2^	*P*	Deviance explained (%)
**δ**^**15**^**N**	Basale	GC	101	1.9	13.4	0.2	**< 0.001**	21.1
	Basale	CCS	120	1.0	8.4	0.6	**< 0.01**	6.7
	Externum	GC	85	1.9	7.4	0.1	**< 0.01**	14.7
	Externum	CCS	63	1.0	5.5	0.1	**< 0.1**	8.3
	Sloughed skin	GC	81	1.8	3.3	0.1	0.7	7.7
	Sloughed skin	CCS	93	1.8	2.6	0.0	0.7	6.7
**δ**^**13**^**C**	Basale	GC	101	1.0	0.2	-0.0	0.7	0.2
	Basale	CCS	120	1.9	3.6	0.1	**< 0.1**	6.2
	Externum	GC	85	1.5	1.3	0.1	0.4	2.8
	Externum	CCS	63	1.5	0.6	0.0	0.6	2.8
	Sloughed skin	GC	81	1.0	1.3	0.0	0.3	1.6
	Sloughed skin	CCS	93	1.0	0.1	-0.0	0.8	0.1

*E*.*df*., Estimated degrees of freedom; F, test of whether the smoothed function significantly reduces model deviance; *P*, p-values in bold were considered statistically significant (<0.05).

The CRD skin δ^15^N values were used as a reference to determine if the isotopic signal of this foraging zone was present in the skin sampled in the GC and the CCS. Some of the observed δ^15^N values in the stratum basale and stratum externum from skin sampled in the CCS could represent transitional values between the CRD isotopic signal and the CCS signal. One of the values observed in the stratum externum sampled in August was assigned to the CRD (Fig 3).

The deviance explained in the relationship between skin δ^15^N values and time for all six GAM models was low (6.7 to 21.1%; Table 2) due to the high degree of dispersion observed in skin data. This degree of variation was expected since the duration of time individual whales had spent in the zone where skin was collected was unknown at the time of sampling. As such, this variation is likely driven by a combination of recently arrived whales that had isotope values reflective of other foraging zones, individuals in the equilibration period with intermediate isotope values that represent a mixture of prey consumed in two foraging zones, or individuals that had reached skin steady-state isotopic equilibrium with the isotopic composition of local prey (Fig 3).

Estimates of δ^15^N isotopic incorporation rate of blue whale skin strata in each foraging zone are shown in Table 3 and S3 Table. In the GC, the stratum basale (81 d), stratum externum (81 d), and sloughed skin (90 d) had similar incorporation rates (Table 3). In the CCS, the stratum basale had longer incorporation rates (262 d) than the stratum externum (192 d). Sloughed skin (272 d) had the lowest isotopic incorporation rate in CCS, although the later estimate had a high degree of uncertainty (Table 3). The average skin strata isotopic incorporation rate in the CCS (242 d) was 158 days lower than the GC (84 d) (Table 3). The overall mean of the δ^15^N isotopic incorporation rate of blue whale skin was estimated, integrating all strata in both zones (163 d, Table 3).

**Table 3:** δ^15^N isotopic incorporation rates of blue whale skin strata in the Gulf of California and California Current System. The number of days were estimated by extrapolating from the GAM predictions (model fit and the upper and lower 95% confidence limits) for skin δ^15^N values to change by 1.6‰ to isotopically equilibrate with local prey in each zone.

Zone	Skin Stratum	δ^15^N isotopic incorporation rate of blue whale skin		
		Model fit	Lower limit	Upper limit
Gulf of California	Basale	81	90	69
	Externum	81	112	69
	Sloughed Skin	90	60	149
**Mean±SD**		**84±5**		
California Current System	Basale	262	222	360
	Externum	192	160	240
	Sloughed Skin	272	163	816
**Mean±SD**		**242±44**		
**Overall Mean±SD**		**163±91**		

**Fig 4:**
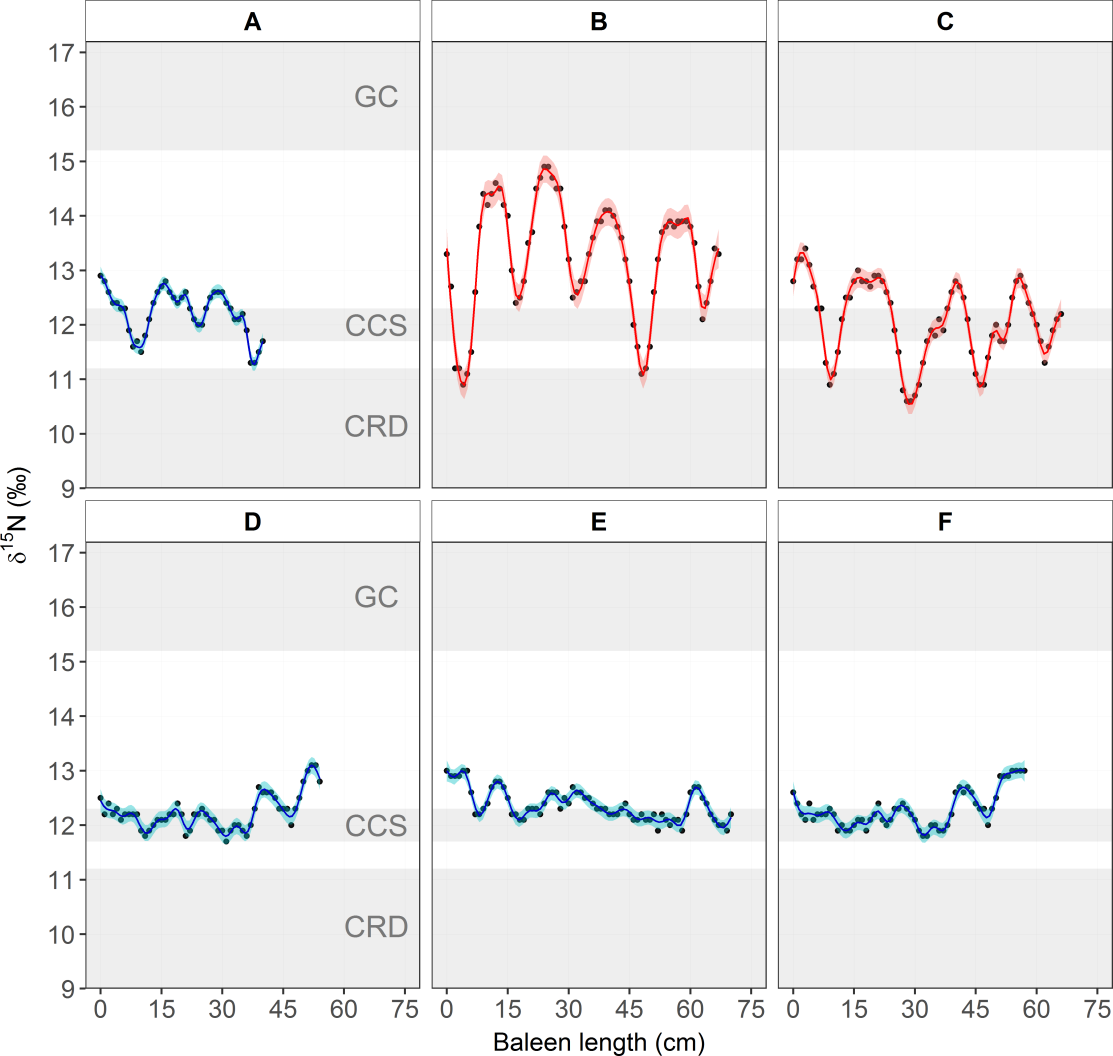
**δ**^**15**^**N values along the baleen plates from six whales, identified as A–F.** Points represent actual values. The continuous line (blue: males; red: females) represents the GAM model fit and the narrow fringe around the lines represent the 95% confidence intervals. The gray shaded area represents the mean±SD of the trophic-corrected blue whale skin values for each foraging zone: Gulf of California (GC), the California Current System (CCS) and the Costa Rica Dome (CRS).

Blue whale baleen isotope values are available in S1 Dataset and S7 Table. Stranding information of baleen plates collected from six blue whales (A to F), is presented in S1 Table and Fig 1. The results of the GAM models to assess the fluctuations in δ^15^N values along baleen plates, and of baleen growth rates estimations are shown in Tables 4 and 5, respectively. The GAM fit showed that the amplitude of the oscillations differed among individuals (Tables 4 and 5, Fig 4). Three baleen plates (A–C, one male and two females; S1 Table) exhibited the expected fluctuations in δ^15^N ranging from 10.6‰ to 14.9‰ (Fig 4A–4C), and the length of baleen between these fluctuations ranged between 13 and 19 cm (Table 5). The other three baleen plates (D–F, all males; S1 Table) maintained relatively constant δ^15^N values, ranging between 11.7‰ and 13.1‰ along the plate (Fig 4D–4F). Inter-individual differences in the amplitude of the oscillations are likely related to the individual migratory strategies and residency time within each foraging zone [37,38,43,55]. By using the trophic-corrected skin δ^15^N values based on that of prey (Table 1), it was possible to associate these oscillations with the potential foraging zone that each individual whale visited. From these data, it could be inferred that whale B moved between all three zones, showing relatively regular cycles (Fig 4B), whereas whale C did not enter the GC, but moved constantly between the CCS and the CRD, in less regular cycles (Fig 4C). Whale A remained mainly within the CCS, potentially only migrating twice to the CRD (Fig 4A). In the case of whales D, E and F, the data suggests that these individuals remained within the CCS, throughout several years (Fig 4D–4F). Only whales A, B, and C were used to estimate the baleen growth rates (Fig 4A–4C). The mean (±SD) growth per year of baleen plates was estimated for each whale (A = 13.5±2.2; B = 14.8±1.7; C = 17.5±1.5 cm y^-1^; Table 5), and also integrated in an overall mean (±SD) (15.5±2.2 cm y^-1^; Table 5).

**Table 4:** GAM results to assess the fluctuations of δ^15^N and δ^13^C in baleen plates.

Isotope	Baleen code	n	*E*.*df*.	F	Adjusted R^2^	*P*	Deviance explained (%)
**δ**^**15**^**N**	A	41	23.8	61.4	1	**< 0.001**	99.0
	B	68	27.2	108.0	1	**< 0.001**	98.7
	C	67	27.3	95.9	1	**< 0.001**	98.6
	D	55	23.3	25.2	0.9	**< 0.001**	95.7
	E	71	26.5	27.2	0.9	**< 0.001**	94.8
	F	58	23.8	31.3	0.9	**< 0.001**	96.3
**δ**^**13**^**C**	A	41	21.9	55.8	1	**< 0.001**	98.8
	B	68	20.3	15.6	0.9	**< 0.001**	89.3
	C	67	27.7	64.9	1	**< 0.001**	98.0
	D	55	24.9	68.4	1	**< 0.001**	98.5
	E	71	27.5	65.1	1	**< 0.001**	97.8
	F	58	25.0	25.1	0.9	**< 0.001**	95.7

*E*.*df*., Estimated degrees of freedom; F, test of whether the smoothed function significantly reduces model deviance; *P*, p-values in bold were considered statistically significant (<0.05).

**Table 5:** Blue whale baleen growth rate: Estimated by using the distance between sequential δ^15^N minimums along the baleen plates from whales A to C.

Baleen code	Sex	Intervals between δ^15^N minimums (cm)	Growth rate (cm y^-1^)
**A**	Male	10–24	14.0
		24–37	13.0
**Mean±SD**			**13.5±0.7**
**B**	Female	4–17	13.0
		17–31	14.0
		31–48	17.0
		48–63	15.0
**Mean±SD**			**14.8±1.7**
**C**	Female	9–27	18.0
		27–46	19.0
		46–62	16.0
**Mean±SD**			**17.5±1.5**
**Overall Mean±SD**			**15.5±2.2**

### δ^13^C values of skin and baleen plates

The mean δ^13^C value of the prey in the GC was 0.7‰ and 2.9‰ higher than the CCS and the CRD, respectively (S2 Table). However, the standard deviation of the CRD overlapped with all the zones and it was not possible to accurately assign the origin of measured δ^13^C from skin nor baleen plates.

The GAM model revealed a very weak though significant positive relationship between the δ^13^C and time for the stratum basale sampled within the CCS. The GAMs applied to the other skin strata, from the other two foraging zones, did not show any relationship between the δ^13^C values and time (Table 2, S1 Fig), and thus the isotopic incorporation rate of δ^13^C in blue whale skin could not be estimated.

Mean (±SD) δ^13^C values of six baleen plates (A–F) are presented in S7 Table. The GAM fits (Table 4, S3 Fig) showed that all individuals presented small oscillations in the δ^13^C values along the baleen that ranged between -18.3 to -16.1. These oscillations could not be linked to the foraging zones because of the overlap in prey δ^13^C among zones (S2 Table). Therefore, baleen growth rates were inferred only using baleen δ^15^N values.

## Discussion

### Influence of lipid-extraction and DMSO preservation on skin δ^13^C and δ^15^N values

Our results suggest that lipid-extraction is necessary to remove biases in skin δ^13^C values associated with lipid content (S4 Table), which agrees with previous studies on mysticetes [75,76]. In regard to the effects of lipid-extraction on δ^15^N values of cetacean skin, some authors [48,75,76] recommend analyzing bulk tissues because lipid-extraction can influence δ^15^N values, although this effect varied between species [75,76] and tissues [75]. In our study, we only compared the δ^15^N of five biopsy samples from which we analyzed paired bulk and lipid-extracted subsamples; however, δ^15^N values between these treatments did not differ significantly, which would be in accordance with the results reported for other marine organisms [95]. With regard to preservation in DMSO (S4 Table), after lipid-extraction, blue whale skin δ^13^C, δ^15^N and C/N ratios of samples preserved in DMSO were similar to those of samples preserved frozen. Our results concur with previous studies that show lipid-extraction via a 2:1 chloroform:methanol solvent solution was a sound method for removing the combined effect that DMSO and tissue lipid content have on skin δ^13^C values [76,81].

### Skin δ^15^N isotopic incorporation rates

Only two studies have estimated isotopic incorporation rates of cetacean skin, and both utilized controlled feeding experiments on captive bottlenose dolphins [47,48]. Our approach was to use gradients in baseline δ^15^N values between the GC and CCS as a natural diet switch experiment (Fig 3). Our mean estimate of δ^15^N isotopic incorporation rates (163±91 d; Table 3) for blue whale skin is similar to that observed in the longest experiment on captive bottlenose dolphins (180±71 d) [48]. The similarity in incorporation rate estimates for these two distantly related cetacean species that differ in weight by over two orders of magnitude is striking, but suggests that these estimates can be applied to other odontocetes and mysticetes.

We found that isotopic incorporation rates varied among skin strata and foraging zones (Table 3); however, all of these estimates fell within the range of those observed for bottlenose dolphins in previous studies (106–275 d and ~60–90 d)[47,48]. It is possible that the observed variation in skin incorporation rates among zones could be influenced by water temperature [10,96–100], with higher rates in the warmer waters of GC in comparison to the CCS (Table 3). In cold waters, marine mammals reduce peripheral blood flow to maintain a constant internal body temperature, which results in a decrease of epidermal metabolism [101–103]. In contrast, incursion into warmer waters accelerates the turnover of superficial skin cells and increase the proliferation rate of cells by intensifying blood flow to the skin stratum basale [104]. Observations suggest that odontocetes, such as belugas (*Delphinapterus leucas*) [104] and killer whales (*Orcinus orca*) [105], move from colder to warmer waters to molt or promote skin regeneration. A study on blue whales in the GC and CCS found that at sites with lower water temperatures, sloughed skin was observed less often in comparison to warmer sites [67].

A novel aproach in this study was to analyze different skin strata: basale, externum, and sloughed skin (Figs 2A and 3). We hypothesised that the different skin strata could provide information about temporal shifts in diet. The stratum basale, where cells are constantly produced, would most likely reflect the most recent dietary information, while the isotopic composition of stratum externum and sloughed skin would record information of the diet consumed in the past, perhaps when individuals were in a different foraging zone than the one where skin samples were collected. The isotopic comparison of strata in the CCS supports this hypothesis since the stratum basale had significantly lower δ^15^N values than the stratum externum and sloughed skin (S5 Table), suggesting that the stratum basale was equilibrating with local prey, characterized by lower δ^15^N values than those which occur in the GC (Table 1, S2 Table). In the GC, skin strata did not have significantly different δ^15^N values; however, sloughed skin had δ^15^N values that were similar to those expected if the skin was grown in the CCS (Table 1, Fig 3), suggesting that sloughed skin samples have a higher probability of providing information about past diets. Thus, skin samples collected from migratory mysticetes can reflect information about past diets independent of where sampling occurs, demonstrating that skin is a valuable tissue to estimate relative contributions of food consumed in different foraging zones utilized during the annual life cycle. Since collecting skin from free ranging cetaceans is cost- and time-intensive, we recommend dividing skin biopsies into strata and collecting sloughed skin when available to increase the amount of information that can be gleaned from isotope analysis of this tissue.

### Baleen growth rates

Our estimate of baleen growth rates for blue whales (~15.5±2.2 cm y^-1^; Table 5) are consistent with previous estimates for other balaenopterids, such as the fin whale (*Balaenoptera physalus*, 20±2.6 cm y^-1^) [37,55], and minke whale (*Balenoptera acutorostrata*, 12.9 cm y^-1^) [57], as well as for other mysticetes such as bowhead whales (16–25 cm y^-1^ in adults) [42,43]. In contrast, baleen growth rate estimates were lower than those for southern right whales (*Eubalaena australis*, ~27 cm y^-1^) [44]. Variation in baleen growth rates among blue whales sampled in this study (Table 4) could be influenced by differences in individual movement strategies (Fig 4A–4C), a hypothesis proposed in previous studies of other mysticete species [37,38,43,55]. For example, variation in the period of time spent within a specific foraging zone or in migration between zones would produce wider or narrower oscillations in baleen δ^15^N, which would influence growth rate estimates (Table 5, Fig 4).

Three of the six baleen plates we analyzed did not show marked oscilations in the δ^15^N values (Fig 4D–4F). These individuals were males: two adults, and one of unknown age class (S1 Table). A potential explanation for a lack of inter-annual variation in δ^15^N is that these whales remained close or within the CCS foraging zone for several years prior to their death. By applying the mean annual growth rate of ~15.5 cm y^-1^ to the baleen records of these three males, they remained within the CCS ecosystems for ~3.5 (Fig 4D), ~4.5 (Fig 4E) and ~3.7 (Fig 4F) years. In contrast, the other three baleen plates, collected from one male and two females, exhibited oscillations in the δ^15^N values along their outer edge that indicate cyclical migrations between foraging zones during ~2.5 (Fig 4A), ~4.3 (Fig 4B) and ~4.2 (Fig 4C) years.

The observed differences in movement strategies of blue whale individuals may be influenced by a combination of the following factors. One general explanation is related to changes in the availability of prey in different foraging zones because it is known that blue whale distribution is influenced by variations in the abundance of their primary prey [2,3]. A more specific explanation is that females are more likely to migrate to warmer waters in winter/spring to nurse their calves, a hypothesis that has been proposed for other mysticetes, although other mysticetes generally do not feed while on their winter/spring breeding grounds [106]. Moreover, the patterns in the baleen of whale C (Fig 4C) suggest a high fidelity of females to returning to specific winter/spring foraging grounds year after year. This would be in accordance with the high site fidelity observed in GC of some well-identified females obtained via photo-identification and genetic analysis [9,11,107]. In the case of males, our data indicate three males remained in the CCS and one migrated twice to the CRD (Fig 4). The female:male sex ratio (1.4:1) in the GC is biased towards females [9,107], suggesting that only a portion of the males in the northeast Pacific are using this zone in winter/spring. Photo-identification data has also shown that some males have a high site fidelity to the GC [9] or possibly other winter/spring foraging grounds. Baleen isotope data from one male in our study also indicates that it had a high fidelity to the CRD, since it migrated only to this zone (Fig 4A). Blue whales are not frequently sighted in the CCS during winter and spring [108,109], although this could be attributed to low search effort during this season. However, vocalizations specific to male blue whales have been recorded year round in the CCS [110–113]. The baleen data of males D, E, F (Fig 4D–4F) is in agreement with this observation. Therefore, we hypothesize that there are two migratory strategies for blue whale males in the northeast Pacific. Some individuals migrate to winter/breeding grounds in the GC or CRD, while others remain within the CCS. How these two migratory strategies influence mating success for males is not known.

### δ^15^N trophic discrimination factors

δ^15^N trophic discrimination factors have not been estimated for blue whale tissues, therefore our approach was to assume a 1.6‰ (Table 1) discrimination factor between whales and their prey based on the controlled feeding experiments on bottlenose dolphins [47,48]. Borrell *et al*. [114] suggested using a trophic discrimination factor of 2.8‰ for balaenopterid skin and baleen plates. However, the mean (±SD) baleen δ^15^N value of the three male blue whales (D: 12.2±0.3; E: 12.4±0.3; F: 12.3±0.4; S7 Table, Fig 4D–4F) that presumably remained within the CCS for ~2–3 years prior to death, and by extension were isotopically equilibrated with local food sources, were enriched by only 1.7–1.9‰ relative to local prey sources (10.5±0.2; S2 Table), and is similar to estimates for skin of captive bottlenose dolphins (1.6±0.5‰) [47,48].

### Temporal consistency of baseline δ^15^N values among foraging zones

The observed seasonal trend in skin δ^15^N values within each zone and the oscillations along baleen plates support our hypothesis that these tissues record baseline shifts in nitrogen isotope values across the northeast Pacific. Our approach assumes that such baseline gradients are temporarily consistent at a decadal scale. To test this assumption, it would be ideal to have prey δ^15^N data from each foraging zone for each year blue whales were sampled; however, such sampling resolution is logistically impossible. Our approach was to use a GLM to evaluate inter-annual trends in skin δ^15^N values, which showed that they slightly increased in the GC and CCS (see Results); no evident trend was observed in the CRD (S6 Table, S4 Fig).

Published datasets show that isotope values of blue whale prey and zooplankton collected from the CCS were consistent over decadal timescales (1994, 2000–2001, 2013) and between sites (Monterey Bay and British Columbia) [60,61,63,64,115]. Moreover, the δ^15^N values in blue whale baleen plates that were assigned to the CCS show a remarkably consistent pattern regardless of when the baleen was collected (1980s vs. 2000s; S1 Table, Fig 4). These patterns suggest that a relatively stable δ^15^N baseline existed in the CCS for nearly three decades. Furthermore, these data suggest that the slight inter-annual increase in skin δ^15^N values of blue whales in the CCS is likely the result of uneven seasonal sampling rather than a shift in the baseline.

δ^15^N values of the dominant krill species (*Nyctiphanes simplex*) in the GC are variable, likely due to their omnivorous feeding behavior [116], but are consistently higher than krill in the CCS and the CRD (S2 Table) [58–62,64,117,118]. Isotope data for potential blue whale prey from the CRD were only available from one study (S2 Table) [62], but zooplankton data also suggest that this zone has lower δ^15^N values in comparison to the CCS and GC [65]. Additionally, baleen δ^15^N patterns from whales that likely visited the CRD (Fig 4A–4C) indicate that baseline δ^15^N values may be consistently lower than those of the other zones. Another factor that may contribute to the observed differences in δ^15^N values among foraging zones is that blue whales in the GC forage on combined aggregations of krill and higher trophic level lanternfish [20]. Thus, blue whale tissues synthetized in the GC will have higher δ^15^N values that result from a combination of baseline and diet factors relative to tissues grown in other foraging zones in the northeast Pacific (Table 1, S3 Table, Figs 3 and 4).

### δ^13^C values in blue whale skin and baleen plates

δ^13^C incorporation rates for skin could not be estimated because of the similarity in δ^13^C values among prey from different foraging zones (S2 Table), and by extension δ^13^C values were not useful to estimate baleen growth rates. Another variable that could contribute to the lack of spatial signal in δ^13^C is movement of blue whales between coastal ^13^C-enriched and ^13^C-depleted oceanic ecosystems [24] within a specific foraging zone [2]. Thus, any latitudinal variation in blue whale skin and baleen δ^13^C values between the CCS, GC, and CRD may be obscured by longitudinal movement between coastal and offshore areas within foraging zones.

## Conclusions

Blue whale skin isotopic incorporation rates and baleen growth rates are similar to other odontocetes and mysticetes, respectively. We recommend collecting skin samples throughout the seasonal residency of migratory mysticetes within specific foraging zones, and dividing skin biopsies into strata. This approach allows for an assessment of seasonal variation in isotope values that could provide insights into movement and/or shifts in seasonal foraging strategies. Furthermore, analyzing both skin and baleen can provide information on the inter-annual variation in prey isotope values within and among foraging zones, as well as provide information about the migratory strategies of individual whales over several years of life, that currently cannot be obtained from satellite telemetry tags that (at best) collect a single year of movement information [2].

## Supporting information

S1 Fig GAM analysis relating skin δ^13^C values to Julian day (presented in months). The points represent the actual δ^13^C values of skin collected from whales within the Gulf of California (open circles) and the California Current System (open triangles). Lines represent the fit (projections) of the GAM model and the fringe around the lines show the 95% confidence intervals.
(TIF)

S2 Fig Sections used from the GAM model predictions to infer δ^15^N isotopic incorporation rates of blue whale skin strata in each foraging zone. The lines represent the GAM model fit (predictions) in the Gulf of California (green) and the California Current System (blue). The fringe around the lines show the 95% confidence intervals. The black dot represents the initial point (*i*.*e*. diet switch) and the red dot the final point of the sections from the predictions that were used from the fit and the lower and upper confidence intervals. Per mil (‰) differences and days passed between points were estimated and then used to extrapolated to a 1.6‰ increase in the Gulf of California, or decrease in California Current System, for skin to reach steady-state isotopic equilibrium with the local prey isotopic signal.
(TIF)

S3 Fig **δ^13^C values along the baleen plates from six whales, identified as A-F.** Points represent actual values, the continuous line (blue: males; red: females) represents the GAM model fit and the fringe around the lines show the narrow 95% confidence intervals.
(TIF)

S4 Fig GLM analysis relating skin δ^15^N values to time (Julian date, presented in years). Points represent the actual δ^15^N values of blue whale skin collected in different zones of the northeast Pacific. Lines represent the fit of the GLM model and the fringe around the lines show the 95% confidence intervals. The gray shaded area represents the meand±SD of the trophic-corrected blue whale skin values for each foraging zone; Gulf of California (GC), California Current System (CCS), and Costa Rica Dome (CRD).
(TIF)

S1 Table Information of baleen plates collected from six blue whales. (DOCX)

S2 Table Mean (±SD) δ^13^C, δ^15^N, and weight percent C/N ratios of potential blue whale prey from each of the three foraging zones in the northeast Pacific. (DOCX)

S3 Table Results from the GAM model sections used to infer δ^15^N isotopic incorporation rates of blue whale skin strata in Gulf of California (GC) and California Current System (CCS). (DOCX)

S4 Table Max-*t test* results comparing the effect of different treatments on skin δ^15^N, δ^13^C and weight percent C/N ratios. (DOCX)

S5 Table Max-*t test* results for the comparison of δ^13^C and δ^15^N values among different skin strata in the Gulf of California (GC) and California Current System (CCS). (DOCX)

S6 Table GLM results relating blue whale skin δ^15^N values to time (Julian date) in the Gulf of California (GC), California Current System (CCS) and Costa Rica Dome (CRD). (DOCX)

S7 Table Mean (±SD) δ^13^C, δ^15^N and weight percent C/N ratios of blue whale baleen plates collected from stranded whales. (DOCX)

S1 Dataset δ^13^C, δ^15^N and weight percent C/N ratios of blue whale skin and baleen plates used in this study. (XLSX)
