# Supplementary figures and images for: Estimating blue whale skin isotopic incorporation rates and baleen growth rates: Implications for assessing diet and movement patterns in mysticetes
Source: PLoS One. 2017 May 31;12(5):e0177880. doi: 10.1371/journal.pone.0177880 (PMC5451050; doi:10.1371/journal.pone.0177880)

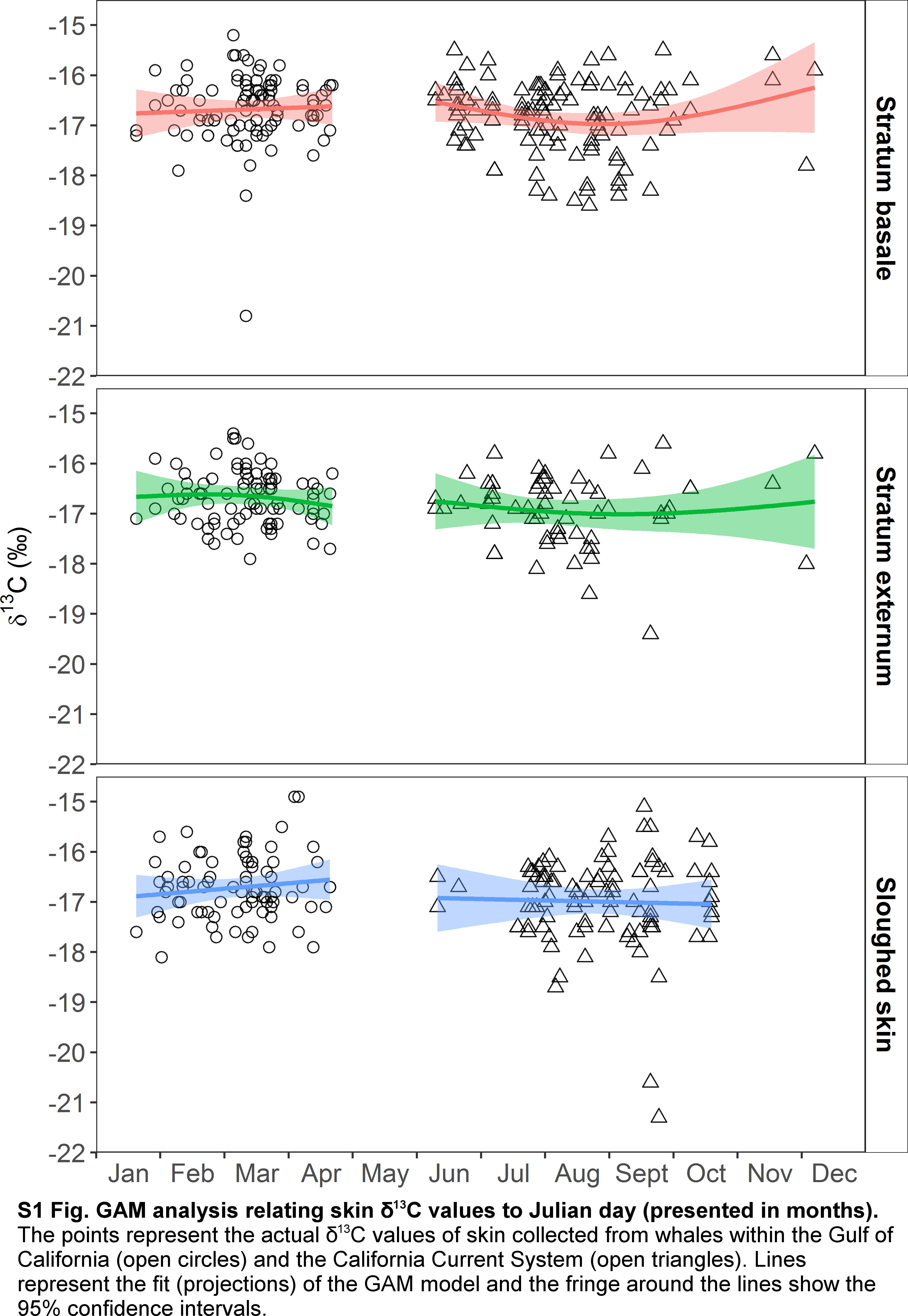

Supplement: S1 Fig — The points represent the actual δ13C values of skin collected from whales within the Gulf of California (open circles) and the California Current System (open triangles). Lines represent the fit (projections) of the GAM model and the fringe around the lines show the 95% confidence intervals. (TIF) [file pone.0177880.s001.tif]

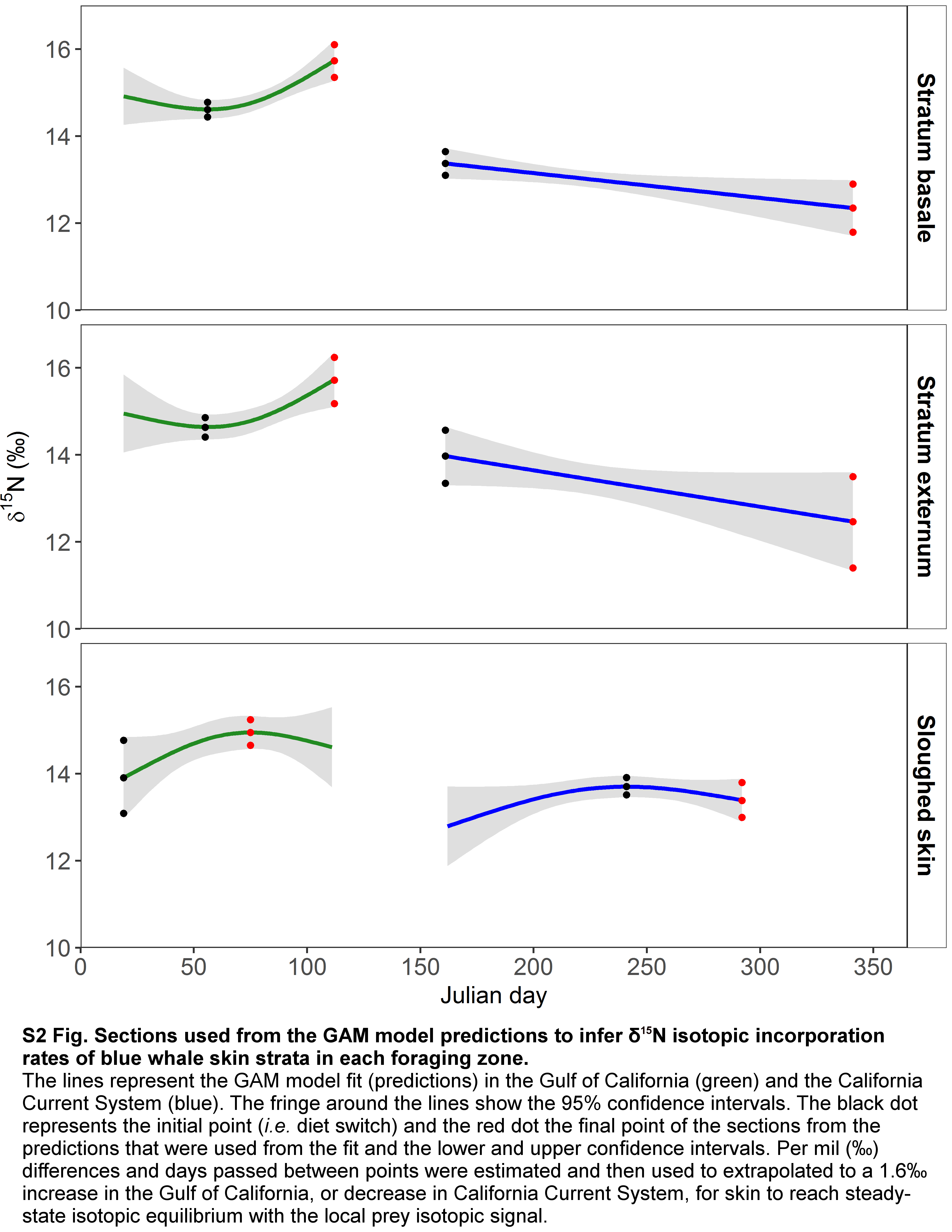

Supplement: S2 Fig — The lines represent the GAM model fit (predictions) in the Gulf of California (green) and the California Current System (blue). The fringe around the lines show the 95% confidence intervals. The black dot represents the initial point (i.e. diet switch) and the red dot the final point of the sections from the predictions that were used from the fit and the lower and upper confidence intervals. Per mil (‰) differences and days passed between points were estimated and then used to extrapolated to a 1.6‰ increase in the Gulf of California, or decrease in California Current System, for skin to reach steady-state isotopic equilibrium with the local prey isotopic signal. (TIF) [file pone.0177880.s002.tif]

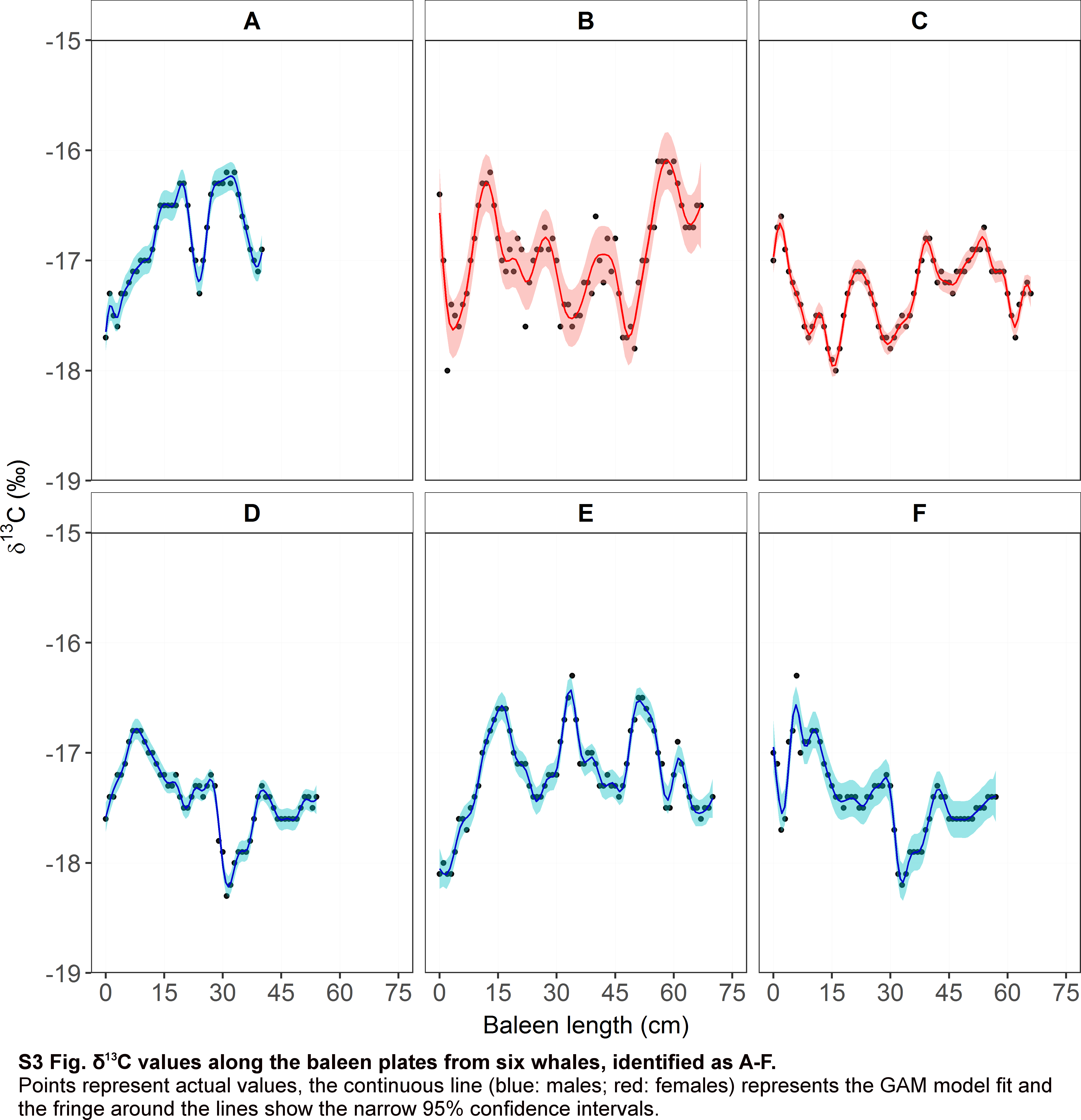

Supplement: S3 Fig — δ13C values along the baleen plates from six whales, identified as A-F. Points represent actual values, the continuous line (blue: males; red: females) represents the GAM model fit and the fringe around the lines show the narrow 95% confidence intervals. (TIF) [file pone.0177880.s003.tif]

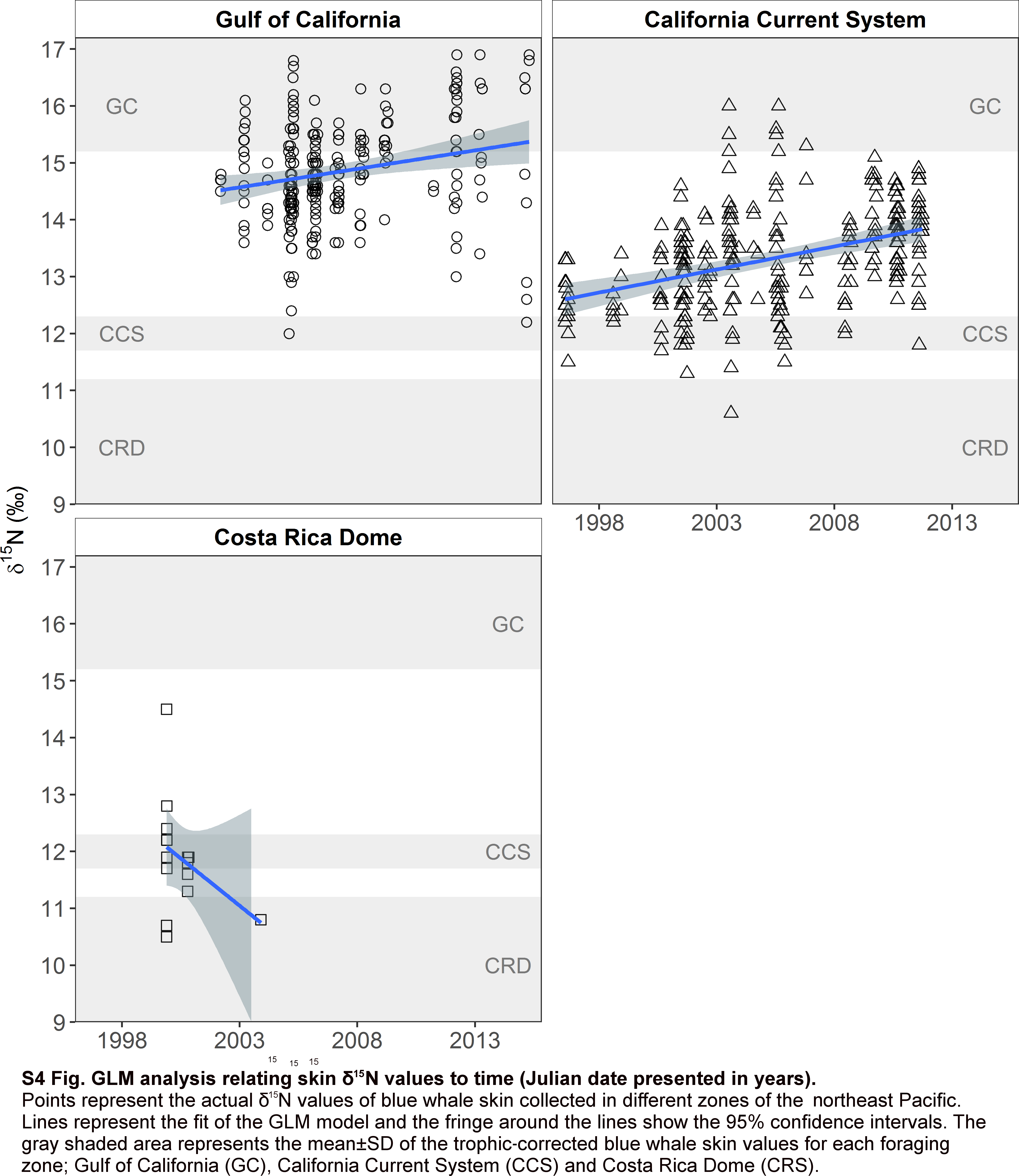

Supplement: S4 Fig — Points represent the actual δ15N values of blue whale skin collected in different zones of the northeast Pacific. Lines represent the fit of the GLM model and the fringe around the lines show the 95% confidence intervals. The gray shaded area represents the meand±SD of the trophic-corrected blue whale skin values for each foraging zone; Gulf of California (GC), California Current System (CCS), and Costa Rica Dome (CRD). (TIF) [file pone.0177880.s004.tif]
