# Supplementary material for: Estimating blue whale skin isotopic incorporation rates and baleen growth rates: Implications for assessing diet and movement patterns in mysticetes
Source: PLoS One. 2017 May 31;12(5):e0177880. doi: 10.1371/journal.pone.0177880 (PMC5451050; doi:10.1371/journal.pone.0177880)
Supplement: S1 Table — (DOCX) [file pone.0177880.s005.docx]

**S1 Table. Information of baleen plates collected from six blue whales.**

| Sample Code | Contributor | Whale Length (m) | Sex | Age Category | Stranding Date | Standing Location | Latitude | Longitude |
| --- | --- | --- | --- | --- | --- | --- | --- | --- |
| A | OMMSN | 21.3 | M | Adult | 01/11/2015 | Oregon, California, USA | 42.5 | -124.4 |
| B | CICIMAR-IPN | 25.9 | F | Adult | 28/03/2007 | Isla Magdalena, BCS, MEX | 25.3 | -112.1 |
| C | HSU-VM | 22.3 | F | Adult | 19/10/2009 | Fort Bragg, California, USA | 39.4 | -123.8 |
| D^*^ | CDPR-PCRSP | ND | M^*^ | ND | ND | West Coast USA | ND | ND |
| E | HSU-VM | 26.5 | M | Adult | 03/09/1988 | Mendocino, California, USA | 37.7 | -122.5 |
| F | HSU-VM | 20.7 | M | Adult | 23/06/1986 | Big Sur, California, USA | 36.3 | -121.9 |

ND, no data.

^*^The stranding data for this baleen plate was unavailable, the sex identity was determined at NOAA-SWFSC by using the genetic methods in Morin *et al.* [71,72].
