# Supplementary material for: Estimating blue whale skin isotopic incorporation rates and baleen growth rates: Implications for assessing diet and movement patterns in mysticetes
Source: PLoS One. 2017 May 31;12(5):e0177880. doi: 10.1371/journal.pone.0177880 (PMC5451050; doi:10.1371/journal.pone.0177880)
Supplement: S2 Table — (DOCX) [file pone.0177880.s006.docx]

**S2 Table. Mean (±SD) δ^13^C, δ^15^N, and weight percent C/N ratios of potential blue whale prey from each of the three foraging zones in the northeast Pacific.**

| Zones | Years | Months | Prey | n | Mean±SD | | | Source |
| --- | --- | --- | --- | --- | --- | --- | --- | --- |
|  |  |  |  |  | **δ^13^C** | **δ^15^N** | **C/N** |  |
| Gulf of California | 2000-2001 | Jan-Apr | *N.s.* | 5 | -17.7±0.1 | 12.6±0.1 | **-** | [117] |
|  | 2004 | Mar | *N.s.* | 1 | -17.8 | 15.0 | 2.9 | This study |
|  | 2005 | Apr | *N.s.* | 7 | -17.9±0.5 | 14.6±0.6 | 3.2±0.1 | This study |
|  | 2006 | Jan-Mar | *N.s.* | 7 | -17.8±1.4 | 13.5±1.6 | 3.0±0.0 | This study |
|  | 2006-2007 | Mar | *N.s.* | 8 | -18.1±0.5 | 14.7±0.8 | - | [118] |
|  | 2013 | Mar | *N.s.* | 3 | -18.2±1.8 | 13.8±1.2 | 3.4±0.0 | This study |
|  | 2014 | Feb | *N.s.* | 5 | -18.1±0.2 | 15.4±1.5 | 3.3±0.2 | This study |
|  | 2015 | Feb, Mar | *N.s.* | 11 | -18.3±0.9 | 14.1±0.8 | 3.3±0.1 | This study |
|  | 2013 | Mar | Lf | 3 | -17±0.3 | 18.1±0.5 | 3.2±0.1 | This study |
|  | 2014 | Feb | Lf | 1 | -16.7 | 18.1 | 3.3 | This study |
|  | 2015 | Feb, Mar | Lf | 3 | -17.7±0.6 | 16.7±1.5 | 3.2±0.1 | This study |
| Mean±SD^a^ |  |  |  |  | **-17.9±0.9** | **14.6±1** |  |  |
| California Current System | 1994 | Feb | *T.s./E.p.* | 5 | -20.2±0.3 | 11.2±0.5 | - | [63] |
|  | 2000 | Aug | *E.p.* | 24 | -20.0±0.1 | 9.7±0.2 | - | [64] |
|  | 2000 | Aug | *T.s.* | 58 | -18.6±0.1 | 11.2±0.1 | - | [64] |
|  | 2002 | Aug | *E.p.* | 11 | -17.9±0.3 | 9.1±0.2 | - | [64] |
|  | 2002 | Aug | *T.s.* | 21 | -16.9±0.3 | 10.1±0.1 | - | [64] |
|  | 2001-2002 |  | *E.p.* | 10 | -19.7±0.8 | 9.9±0.4 | - | [59] |
|  | 2002 |  | *T.s.* | 5 | -18.6±2 | 11.1±0.8 | - | [60] |
|  | 2013 |  | *T.s.* | 10 | -17.3±0.4 | 9.6±0.6 | - | [61] |
| Mean±SD^a^ |  |  |  |  | **-18.6±0.4** | **10.4±0.3** |  |  |
| Costa Rica Dome | 2007-2008 | Nov-Dec | Krill | 14-15 | -20.8±2^b^ | 8.5±1.1 | 5.8±0.2 | [62] |
| Mean±SD |  |  |  |  | **-20.8±2^b^** | **8.5±1.1** |  |  |

*N.s, Nyctiphanes simplex*; Lf, Lanterfish; *T.s.*, *Thysanoesa spinifera*; *E.p*., *Euphausia pacifica*

^a^ The mean±sd isotope values of the prey from these zones were estimated by pooling the means and variances of the data from this study and from the literature.

^b^ Krill samples used in that study were not lipid-extracted. Williams [62] provided a mean weight percent C/N ratio of 5.8 for bulk krill samples. If krill δ^13^C data from the Costa Rica Dome is lipid-normalized using this C/N ratio and equations in McConnaughey and McRoy [74], the mean δ^13^C value would be -19.4‰ and there would be greater overlap in δ^13^C values among prey of different zones (Costa Rica Dome, California Current System and Gulf of California).
