# Supplementary material for: Estimating blue whale skin isotopic incorporation rates and baleen growth rates: Implications for assessing diet and movement patterns in mysticetes
Source: PLoS One. 2017 May 31;12(5):e0177880. doi: 10.1371/journal.pone.0177880 (PMC5451050; doi:10.1371/journal.pone.0177880)
Supplement: S3 Table — (DOCX) [file pone.0177880.s007.docx]

**S3 Table. Results from the GAM model sections used to infer δ^15^N isotopic incorporation rates of blue whale skin strata in Gulf of California (GC) and California Current System (CCS).**

| Zone | Strata | Model section | Initial δ^15^N  (diet switch) | Final δ^15^N | Per mil differences (‰) | Days passed between initial and final δ^15^N | Extrapolate to a 1.6‰ change |
| --- | --- | --- | --- | --- | --- | --- | --- |
| GC | Basale | Fit | 14.6 | 15.7 | 1.1 | 56 | 81 |
|  | Basale | Upper 95% CI | 14.8 | 16.1 | 1.3 | 56 | 69 |
|  | Basale | Lower 95% CI | 14.4 | 15.4 | 1.0 | 56 | 90 |
|  | Externum | Fit | 14.6 | 15.7 | 1.1 | 56 | 81 |
|  | Externum | Upper 95% CI | 14.9 | 16.2 | 1.3 | 56 | 69 |
|  | Externum | Lower 95% CI | 14.4 | 15.2 | 0.8 | 56 | 112 |
|  | Sloughed skin | Fit | 13.9 | 14.9 | 1.0 | 56 | 90 |
|  | Sloughed skin | Upper 95% CI | 14.6 | 15.2 | 0.6 | 56 | 149 |
|  | Sloughed skin | Lower 95% CI | 13.2 | 14.7 | 1.5 | 56 | 60 |
|  |  |  |  |  |  |  |  |
| CCS | Basale | Fit | 13.4 | 12.3 | -1.1 | 180 | 262 |
|  | Basale | Upper 95% CI | 13.6 | 12.8 | -0.8 | 180 | 360 |
|  | Basale | Lower 95% CI | 13.1 | 11.8 | -1.3 | 180 | 222 |
|  | Externum | Fit | 14.0 | 12.5 | -1.5 | 180 | 192 |
|  | Externum | Upper 95% CI | 14.5 | 13.3 | -1.2 | 180 | 240 |
|  | Externum | Lower 95% CI | 13.4 | 11.6 | -1.8 | 180 | 160 |
|  | Sloughed skin | Fit | 13.7 | 13.4 | -0.3 | 51 | 272 |
|  | Sloughed skin | Upper 95% CI | 13.9 | 13.8 | -0.1 | 51 | 816 |
|  | Sloughed skin | Lower 95% CI | 13.5 | 13.0 | -0.5 | 51 | 163 |

CI, confidence interval limit
