# Supplementary material for: Estimating blue whale skin isotopic incorporation rates and baleen growth rates: Implications for assessing diet and movement patterns in mysticetes
Source: PLoS One. 2017 May 31;12(5):e0177880. doi: 10.1371/journal.pone.0177880 (PMC5451050; doi:10.1371/journal.pone.0177880)
Supplement: S4 Table — (DOCX) [file pone.0177880.s008.docx]

**S4 Table. Max-*t* test results comparing the effect of different treatments on skin δ^15^N, δ^13^C and weight percent C/N ratios.**

| Variable | Mean±SD (n) | | Treatment comparison | Diff | CI: 95% | SE | *t* | *P* |
| --- | --- | --- | --- | --- | --- | --- | --- | --- |
|  | **Bulk skin** | **Lipid extracted** |  |  |  |  |  |  |
| δ^15^N | 14.5±0.3 (5) | 14.6±0.3 (5) | Bulk skin - Lipid extracted | -0.1 | -0.6 ‒ 0.4 | 0.2 | -0.4 | 0.7 |
| δ^13^C | -18.4±0.4 (5) | -16.5±0.1 (5) | Bulk skin - Lipid extracted | -1.9 | -2.3 ‒ -1.5 | 0.2 | -10.4 | **<0.001** |
| C/N ratio | 4.2±0.1 (5) | 3.2±0.0 (5) | Bulk skin - Lipid extracted | 1.0 | 0.8 ‒ 1.2 | 0.1 | 12.9 | **<0.001** |
|  | | | | | | | | |
|  | **Mean±SD (n)** | |  |  |  |  |  |  |
|  | **Frozen/LE** | **DMSO/LE** |  |  |  |  |  |  |
| δ^15^N | 14.0±0.9 (25) | 13.9±0.9 (25) | Frozen/LE - DMSO/LE | 0.1 | -0.5 ‒ 0.6 | 0.2 | 0.2 | 0.8 |
| δ^13^C | -16.9±0.6 (25) | -16.9±0.5 (25) | Frozen/LE - DMSO/LE | 0.0 | -0.3 ‒ 0.4 | 0.2 | 0.2 | 0.8 |
| C/N ratio | 3.0±0.2 (25) | 3.0±0.2 (25) | Frozen/LE - DMSO/LE | -0.0 | -0.1 ‒ 0.1 | 0.1 | -0.4 | 0.7 |

LE, lipid-extracted skin; Diff, estimated differences between group means; CI, confidence intervals; SE, Standard error; *t*, test value; *P*, adjusted p values reported, values in bold were considered statistically significant (<0.05).
