# Supplementary material for: Estimating blue whale skin isotopic incorporation rates and baleen growth rates: Implications for assessing diet and movement patterns in mysticetes
Source: PLoS One. 2017 May 31;12(5):e0177880. doi: 10.1371/journal.pone.0177880 (PMC5451050; doi:10.1371/journal.pone.0177880)
Supplement: S5 Table — (DOCX) [file pone.0177880.s009.docx]

**S5 Table. Max-*t test* results for the comparison of δ^13^C and δ^15^N values among different skin strata in the Gulf of California (GC) and California Current System (CCS).**

| Zone | Isotope | Mean±SD (n) | | | Strata comparison | Diff | CI: 95% | SE | *t* | *P* |
| --- | --- | --- | --- | --- | --- | --- | --- | --- | --- | --- |
|  |  | **Stratum Basale** | **Stratum Externum** | **Sloughed Skin** |  |  |  |  |  |  |
| GC | δ^15^N | 14.9±0.7 (101) | 14.9±0.8 (85) | 14.7±1.0 (81) | Basale - Sloughed skin | 0.2 | -0.2‒0.5 | 0.1 | 1.1 | 0.5 |
|  |  |  |  |  | Externum - Sloughed skin | 0.2 | -0.2‒0.5 | 0.2 | 1.1 | 0.5 |
|  |  |  |  |  | Externum - Basale | 0.0 | -0.2‒0.3 | 0.1 | 0.1 | 1 |
|  | δ^13^C | -16.7±0.7 (101) | -16.7±0.5 (85) | -16.7±0.6 (81) | Basale - Sloughed skin | 0.0 | -0.2‒0.3 | 0.1 | 0.4 | 0.9 |
|  |  |  |  |  | Externum - Sloughed skin | 0.1 | -0.2‒0.3 | 0.1 | 0.5 | 0.9 |
|  |  |  |  |  | Externum - Basale | 0.0 | -0.2‒0.2 | 0.1 | 0.1 | 1 |
| CCS | δ^15^N | 13±0.8 (120) | 13.4±1.1 (63) | 13.6±0.7 (93) | Basale - Sloughed skin | -0.5 | -0.8 ‒ -0.3 | 0.1 | -4.9 | **<0.001** |
|  |  |  |  |  | Externum - Sloughed skin | -0.1 | -0.5‒0.2 | 0.2 | -0.7 | 0.7 |
|  |  |  |  |  | Externum - Basale | 0.4 | 0.0‒0.8 | 0.2 | 2.6 | **<0.001** |
|  | δ^13^C | -16.8±0.7 (120) | -16.9±0.7 (63) | -17±0.9 (93) | Basale - Sloughed skin | 0.2 | -0.1‒0.4 | 1.4 | 1.4 | 0.3 |
|  |  |  |  |  | Externum - Sloughed skin | 0.0 | -0.2‒0.4 | 0.5 | 0.5 | 0.9 |
|  |  |  |  |  | Externum - Basale | -0.1 | -0.3‒0.1 | -0.9 | -0.9 | 0.6 |

Diff, estimated differences between group means; CI, confidence intervals; SE, Standard error; *t*, test value; *P*, adjusted p values reported, values in bold were considered statistically significant (<0.05).
