# Supplementary material for: Estimating blue whale skin isotopic incorporation rates and baleen growth rates: Implications for assessing diet and movement patterns in mysticetes
Source: PLoS One. 2017 May 31;12(5):e0177880. doi: 10.1371/journal.pone.0177880 (PMC5451050; doi:10.1371/journal.pone.0177880)
Supplement: S6 Table — (DOCX) [file pone.0177880.s010.docx]

**S6 Table. GLM results relating blue whale skin δ^15^N values to time (Julian date) in the Gulf of California (GC), California Current System (CCS) and Costa Rica Dome (CRD).**

| Zone | n | Model | Coefficient ± SME | CI (95%) | *t* | Residual deviance | df | AIC | *P* |
| --- | --- | --- | --- | --- | --- | --- | --- | --- | --- |
| GC | 267 | δ^15^N_skin_ =12.4 + 1.8e^-4^·time^a^ | α = 12.4 ± 0.6  β = 1.8e^-4^ ± 4.7e^-5^ | 11.2 – 13.7  8.5e^-5^ – 3.0e^-4^ | 3.8 | 190.6 | 265 | 673.8 | **< 0.001** |
| CCS | 276 | δ^15^N_skin_ =10.5 + 2.2e^-4^·time^a^ | α = 10.5 ± 0.4  β = 2.2e^-4^ ± 3.0e^-5^ | 9.7 – 11.2  2.0e^-4^ – 2.8e^-4^ | 7.3 | 184.9 | 274 | 678.7 | **< 0.001** |
| CRD | 16 | δ^15^N_skin_ =22 - 9.1e^-4^·time^a^ | α = 22 ± 7.0  β = -9.1e^-4^ ± 6.3e^-4^ | 8.2 – 35.8  -2.0e^-3^ – 3.3e^-4^ | -1.4 | 11.4 | 14 | 46.0 | 0.2 |

α, intercept parameter; β, slope parameter; SME, standard error of the mean; CL, confident intervals of the mean; *t*, test values; *P*, p values reported, values in bold were considered statistically significant (<0.05).

**^a^** time, in the model represents number of days.
