# Supplementary material for: Estimating blue whale skin isotopic incorporation rates and baleen growth rates: Implications for assessing diet and movement patterns in mysticetes
Source: PLoS One. 2017 May 31;12(5):e0177880. doi: 10.1371/journal.pone.0177880 (PMC5451050; doi:10.1371/journal.pone.0177880)
Supplement: S7 Table — (DOCX) [file pone.0177880.s011.docx]

**S7 Table. Mean (±SD) δ^13^C, δ^15^N and weight percent C/N ratios of blue whale baleen plates collected from stranded whales.**

| Baleen code | Sex | n | Mean±SD | | |
| --- | --- | --- | --- | --- | --- |
|  |  |  | **δ^13^C** | **δ^15^N** | **C/N** |
| A | M | 40 | -16.8±0.4 | 12.2±0.4 | 3.4±0.1 |
| B | F | 67 | -17.0±0.5 | 13.3±1.1 | 3.6±0.1 |
| C | F | 66 | -17.3±0.3 | 12.1±0.7 | 3.5±0.0 |
| D | M | 54 | -17.4±0.3 | 12.2±0.3 | 3.5±0.1 |
| E | M | 70 | -17.2±0.4 | 12.4±0.3 | 3.5±0.0 |
| F | M | 57 | -17.4±0.4 | 12.3±0.4 | 3.5±0.1 |
